# Fractional flow reserve and instantaneous wave-free ratio in coronary artery bypass grafting: a meta-analysis and practice review

**DOI:** 10.3389/fcvm.2024.1348341

**Published:** 2024-03-07

**Authors:** R. G. Abbasciano, G. R. Layton, S. Torre, N. Abbaker, A. Copperwheat, C. Lucarelli, S. Bhandari, S. Nijjer, G. Mikhail, R. Casula, M. Zakkar, A. Viviano

**Affiliations:** ^1^ Department of Cardiothoracic Surgery, Imperial College Healthcare NHS Trust, London, United Kingdom; ^2^ Department of Cardiovascular Sciences, University of Leicester, Leicester, United Kingdom; ^3^ Department of Cardiac Surgery, University Hospitals of Leicester NHS Trust, Leicester, United Kingdom; ^4^ Cardiac Surgery Unit, Giaccone Hospital, Palermo, Italy; ^5^ Department of Cardiology, University Hospitals of Leicester NHS Trust, Leicester, United Kingdom

**Keywords:** coronary artery bypass grafting, coronary disease, fractional flow reserve, instantaneous wave-free ratio, myocardial infarction, functional ischaemia

## Abstract

**Objective:**

Fractional flow reserve (FFR) and instantaneous wave-free ratio (iFR) are invasive methods to assess the functional significance of intermediate severity coronary lesions. Both indexes have been extensively validated in clinical trials in guiding revascularisation in patients with stable ischaemic heart disease undergoing percutaneous coronary intervention (PCI) with improved clinical outcomes. However, the role of these tools in coronary artery bypass grafting (CABG) is less clear.

**Methods:**

A meta-analysis of randomised trials and observational studies was carried out to help in determining the optimal strategy for assessing lesion severity and selecting graft targets in patients undergoing CABG. Electronic searches were carried out on Embase, MEDLINE, and Web of Science. A group of four authors independently screened and then assessed the retrieved records. Cochrane's Risk of Bias and Robins-I tools were used for bias assessment. A survey was conducted among surgeons and cardiologists to describe current attitudes towards the preoperative use of functional coronary investigations in practice.

**Results:**

Clinical outcomes including mortality at 30 days, perioperative myocardial infarction, number of grafts, incidence of stroke, rate of further need for revascularisation, and patient-reported quality of life did not differ in CABG guided by functional testing from those guided by traditional angiography.

The survey revealed that in half of the surgical and cardiology units functional assessment is performed in CABG patients; there is a general perception that functional testing has improved patient care and its use would clarify the role of moderate coronary lesions that often need multidisciplinary rediscussions; moderate stenosis are felt to be clinically relevant; and anatomical considerations need to be taken into account together with functional assessment.

**Conclusions:**

At present, the evidence to support the routine use of functional testing in intermediate lesions for planning CABG is currently insufficient. The pooled data currently available do not show an increased risk in mortality, myocardial injury, and stroke in the FFR/iFR-guided group. Further trials with highly selected populations are needed to clarify the best strategy.

**Systematic Review Registration:**

ClinicalTrials.gov, identifier (CRD42023414604).

## 1 Introduction

Fractional flow reserve (FFR) and instantaneous wave-free ratio (iFR) are pressure-derived invasive physiology indexes that measure the functional significance of coronary lesions (1). FFR is a measurement of the pressure difference across a coronary artery stenosis during maximal hyperaemia, which is induced by the administration of a vasodilator such as adenosine or papaverine (2), and is usually calculated by dividing the distal coronary pressure by the proximal aortic pressure during the entire cardiac cycle with a calculated range from 0 to 1. It is accepted that an FFR value of less than 0.80 indicates a hemodynamically significant stenosis (3). The iFR on the other hand is a more novel non-hyperaemic index that measures the ratio of the pressure gradient across a coronary artery stenosis during a specific period of the diastole, namely, the wave-free period. During the wave-free period, intracoronary resistance is naturally at its minimum, and the coronary pressure and blood flow are proportional and stable, without fluctuations (4). The iFR is calculated by dividing the distal coronary pressure by the proximal aortic pressure during the wave-free period, and it ranges from 0 to 1 with a value less than 0.89 indicating a hemodynamically significant stenosis (5, 6).

Functional testing indexes have been extensively validated in clinical trials, and their use has been associated with improved clinical outcomes in patients undergoing percutaneous intervention (PCI) in the setting of intermediate grade coronary stenoses (40%–90%), and in multivessel coronary artery disease (CAD) (6–10).

Although these tools are commonly used to guide PCI (11), their role in coronary artery bypass grafting (CABG) is less clear (12, 13). Moreover, there are no guidelines clearly outlining if or when functional coronary physiology indexes should be used before coronary surgery and so implementation appears to be largely unit dependent; their role therefore remains contentious, with the population of which patients may benefit from its use being poorly defined. Accordingly, we conducted an international, electronic cross-sectional sampling of adult interventional cardiologists and cardiac surgeons to establish their opinions on this matter to aid interpretation of this review in the context of current opinion from many specialists and not only those of the authors.

## 2 Materials and methods

### 2.1 Systematic review

A systematic review was performed based on the methods described in the Cochrane Handbook for Systematic Reviews of Interventions (14). The study protocol was registered prospectively and is available under the International Prospective Register of Systematic Reviews (PROSPERO) record CRD42023414604. The study was reported as per the Preferred Reporting Items for Systematic Reviews and Meta-Analyses (PRISMA) statement (15).

Electronic searches for the studies were carried out on Embase, MEDLINE, and Web of Science from database inception to June 2023, using a combination of MeSH terms and key words in subject fields relating to invasive coronary physiology assessments and coronary artery bypass graft surgery (the search strategy is available in the Supplementary Material). We restricted the search strategy to randomised control trials (RCTs) and observational studies with a control group. No restrictions based on language were applied. We checked the reference list of retrieved studies for eligible trials. To identify relevant ongoing trials, an additional search was conducted on ClinicalTrials.gov and International Clinical Trials Registry Platform (ICTRP).

We included RCTs and retrospective or observational studies conducted on adults (age 18 years old or over) undergoing elective or emergency isolated coronary artery bypass grafting, in which FFR or iFR was performed during the preoperative angiographic study and used to guide revascularisation. We excluded trials in which a combined surgical treatment of valvular disease was performed or in which there was no available control group for patients that did not receive the intervention.

The primary outcomes of this review were mortality (at longest available follow-up) and perioperative myocardial infarction. We also assessed the following secondary outcomes: number of grafts, stroke (during hospital stay), kidney injury (during hospital stay), length of stay in the intensive care unit, rate of repeat unplanned revascularisation (at the longest follow-up available), and patient-reported quality of life (QoL) (at the longest follow-up available).

Three reviewers (GL, RA, and CL) identified studies for inclusion by independently looking at titles and abstracts. The full text of the selected studies was then retrieved and assessed for inclusion. Any conflicts were resolved by group discussions. The references were collected and managed using the web-tool Rayyan (16). Data were extracted by four independent reviewers (ST, NA, AC, and RA) onto a purpose-designed data collection spreadsheet using Microsoft Excel [Microsoft Corporation. Microsoft Excel (Internet). 2018. Available from: https://office.microsoft.com/excel].

The extracted data included: year, language and country of publications, sample size, participant demographics, baseline comorbidities, nature of interventions (FFR, iFR, and adopted cut-off), specifics of the surgical technique adopted (arterial grafts used, on-pump/off-pump CABG), outcomes. Approximately 10% of the search was cross validated to control for inter-assessor variability. The reviewers independently assessed the risk of bias in all the trials included in accordance with the guidelines detailed in the Cochrane Handbook for Systematic Reviews, Risk of bias (RoB Tool v1) as low, high, or uncertain risk. A sensitivity analysis was planned in trials at low risk of bias in all domains (17). The Newcastle–Ottawa scale was used for the assessment of bias in observational studies (18).

For categorical variables, the risk ratio (RR) with 95% confidence interval (CI) was calculated, while for continuous variables, we reported the mean difference (MD) with 95% CI. For any parameter using a different scale, the standardised mean difference (SMD) with 95% CI was calculated.

When feasible, an intention-to-treat analysis was conducted on the results. For any categorical data presented as a percentage, the frequency was estimated using the reported sample size and study population.

Meta-analyses were performed in accordance with the recommendations of the Cochrane Handbook for Systematic Reviews of Intervention using the R software package (R Foundation for Statistical Computing, Vienna, Austria) (19, 20).

For the primary analysis, the results of a random-effects model were compared with the fixed-effects model to assess the small study effects. For continuous outcomes, pooled mean differences or standardised mean differences were analysed by using the inverse variance method.

We used the *I*^2^ statistic to measure heterogeneity among the studies in each analysis, although we acknowledge that there is substantial uncertainty in the value of *I*^2^ when there are only a small number of studies. In case of substantial or considerable heterogeneity (*I*^2^ above 60%), we planned to explore possible causes by prespecified subgroup analysis (for primary outcomes only).

#### 2.1.1 Expert opinion survey

Healthcare professionals working within adult cardiac surgery and cardiology were invited to participate in this cross-sectional convenience sampling survey through the mailing list of the British Cardiovascular Society (21), the Society for Cardiothoracic Surgery in Great Britain and Ireland (22), and the British Junior Cardiologist's Association (23) between 1June and 31August 2023. The survey was closed on 15 September 2023. The survey (Supplementary Appendix A) was designed to gather a cross-sectional convenience sampling of opinions to guide discussion points within this work. The consensus of opinions was predefined as a view being shared by at least 60% of responders for a given questionnaire item. Missing data, such as no responses provided to a question, were handled by exclusion.

Survey questions were tailored to a surgical or physician background depending upon the responder but focused upon the same content. All questions were asked in the specific context of patients being considered for CABG surgery. For simplicity of presentation, in this paper we describe all responders working in cardiac surgery as “surgeons” and all those in cardiology as “cardiologists.”

## 3 Results

### 3.1 Meta-analysis

**Figure 1:**
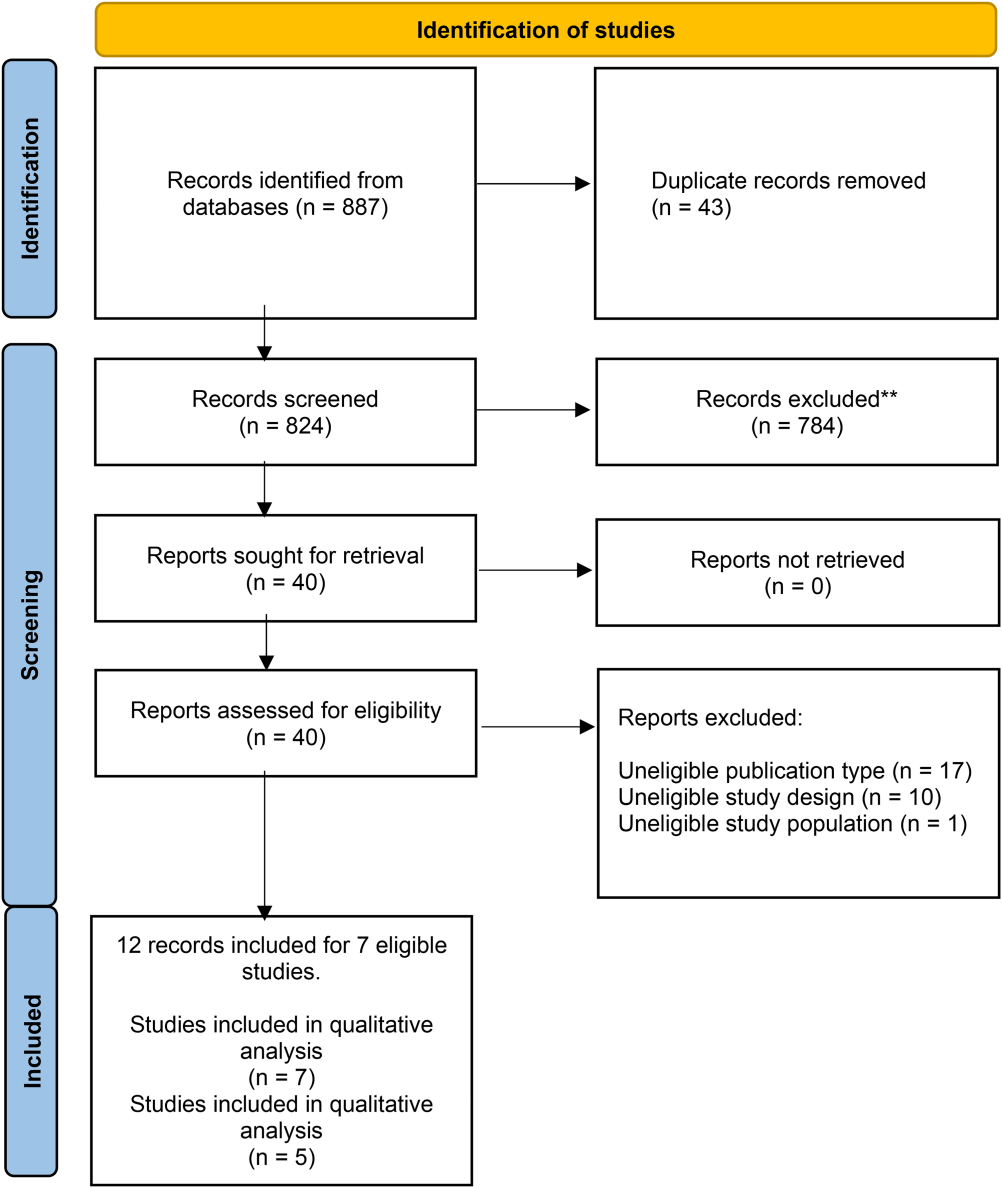
PRISMA diagram for included studies. *From:* Page MJ, McKenzie JE, Bossuyt PM, Boutron I, Hoffmann TC, Mulrow CD, et al. The PRISMA 2020 statement: an updated guideline for reporting systematic reviews. BMJ 2021;372:n71. doi: 10.1136/bmj.n71.

A total of 887 abstracts were retrieved from the searches (Figure 1). After removing duplicate entries, 824 articles were screened, and 784 articles were excluded on the basis of titles and abstracts; a total of 40 relevant publications were retrieved for further assessment. Seven studies (24–30) were included in the qualitative analysis, while five studies (three RCT and two observational studies), analysing a total of 2032 participants, met the inclusion criteria and were included in the quantitative analysis.

A summary of the characteristics of the studies included is reported in Table 1. There was no disagreement among the reviewers as to the selection of the studies. All studies were conducted on a standard-risk population as defined by the study authors. The functional test adopted was FFR in six studies (86%) and a combination of iFR and FFR in one study (14%).

**Table 1:** Characteristics of participants in the included studies.

Study ID	Type of study	Unit of analysis	Complexity	Reported follow-up duration	Age mean (ffr)	Age mean (control)	Males (ffr) (%)	Males (control) (%)	Diabetes (ffr) (%)	Diabetes (control) (%)	PVD (ffr) (%)	PVD (control) (%)	CKD (ffr) (%)	CKD (control) (%)	BMI mean (ffr)	BMI mean (control)
Botman et al. 2007 (24)	Observational	Grafts	Single vessel disease (n = 1) Two-vessels disease (n = 46) Three-vessels disease (n = 107)	12 months	62.4		48									
Fournier et al. 2018 (25)	Observational	Patients	* *	Median 85 months (IQR 66-104)	66	66	82	79	21	22	14	16			28	28
Glineur et al. 2019 (26)	Observational	Grafts	Mean syntax score: 22.9 (functional group); 21.1 (angiography group)	Mean 6.6 months (SD 0.9)												
Moscona et al. 2018 (27)	Observational	Patients	Multivessel disease: n = 90 (angiography); n = 13 (functional)	18 months	58.70	63.80	64	76	57	44	0	14			32.10	28.80
FUTURE	RCT	Patients	Mean syntax score: 19 ± 8 (functional group); 18 ± 8 (angiography group)	Median 24 months (IQR 12.5-37.1)	65	66	84	84	31	32			40	39	28	27
FARGO	RCT	Patients	3 vessels-disease: Functional—39 (78%) Angiography—34 (71%)	6 months	66.4	65.3	90	92	22	23					27.7	27.4
GRAFFITI	RCT	Patients	median of three lesions per patient	12 months	67	67	83	79	35	39						

The evidence for IFR-based strategies in surgical revascularisation is insufficient and thus its role could not be assessed with adequate confidence.

With the exception of Glineur (2019) (routine total arterial revascularisation) and Fournier (2018) (average of two arterial conduits with interquartile range between 1 and 2 for all patients), the specifics related to the adoption of arterial conduits is sparsely reported. The rate of off-pump operations was available for three studies (Fournier 2018, FARGO, and GRAFFITI), and it was 30% in the functional test cohorts and 24% in the angiogram-guided group.

Duration and type of follow-up was heterogeneous between all studies (see Table 1). The shortest reported follow-up duration was 6 months, with the longest a median duration of 85 months (IQR 66–104 months). Five studies utilised angiographic follow-up (26, 29–32) although loss to follow-up or patients non-consenting to repeat angiography were generally high. All studies, except that of Glineur (26) who used angiography in isolation, undertook clinical follow-up evaluating symptomology and quality of life.

**Figure 2:**
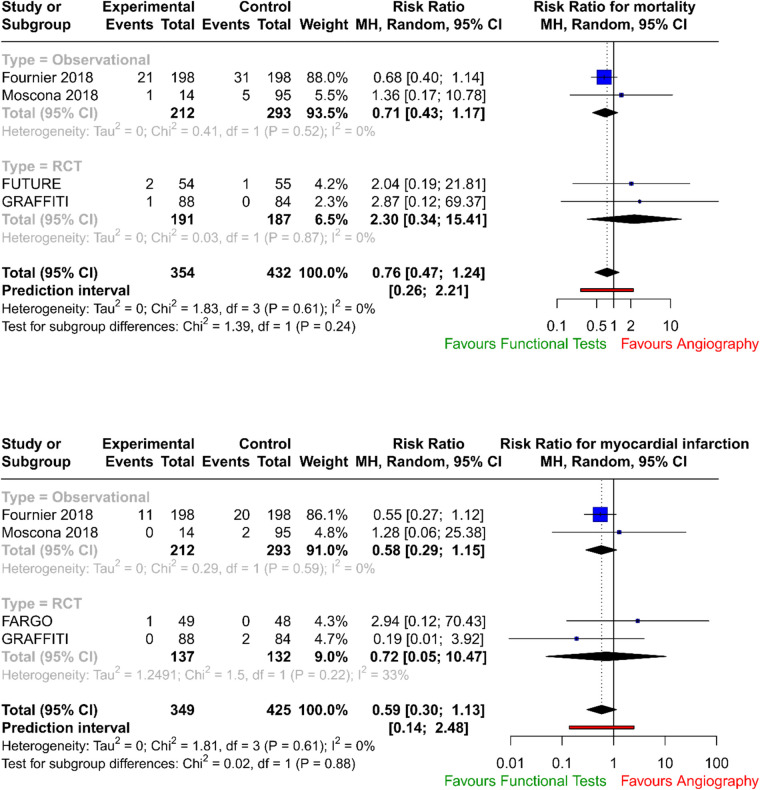
Forest plots related to the analysis of the primary outcomes.

**Figure 3:**
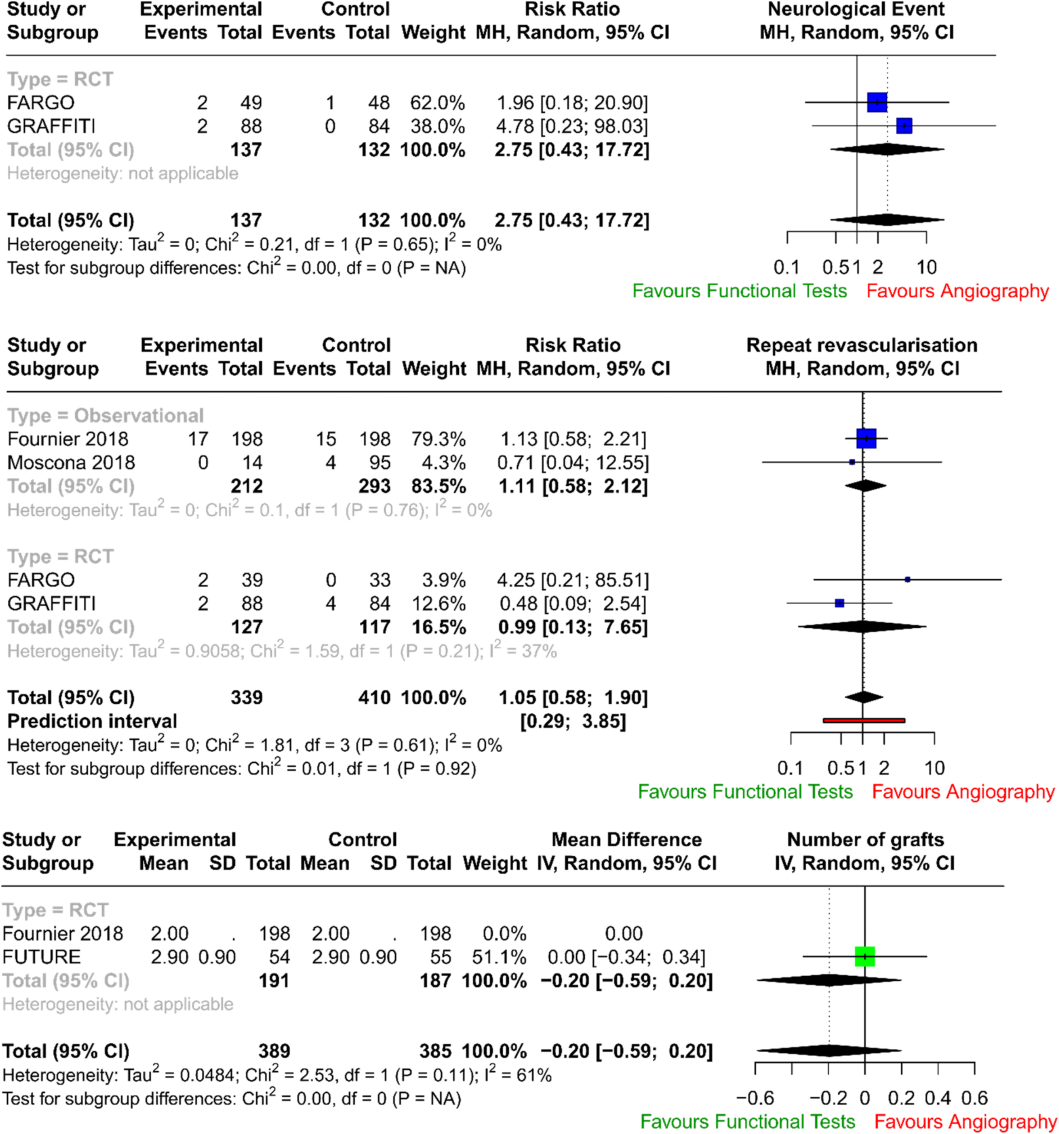
Forest plots related to the analysis of the secondary outcomes.

The forest plots with pooled estimates from random-effects meta-analyses are reported in Figure 2 (primary outcomes), Figure 3 (secondary outcomes) and in the Supplementary Material. In the primary analysis, when compared with conventional angiography, functional testing to guide CABG had no clear effect on mortality (four studies; RR 0.76; 95% CI: 0.47–1.24; *I*^2^ = 0%) and on myocardial injury (four studies; RR 0.59; 95% CI: 0.30–1.13; *I*^2^ = 0%). There was no clear effect on secondary outcomes such as postoperative neurological events (two trials; RR 2.75; 95% CI: 0.43–17.72; *I*^2^ = 0%), number of grafts (two trials; MD, −0.20; 95% CI: −0.59 to 0.20; *I*^2^ = 61%), and need for repeat unplanned revascularisation (four trials; RR 1.05; 95% CI: 0.58–1.90; *I*^2^ = 0%). There were no available data to assess the effect of preoperative functional testing on renal injury. The investigators from FARGO reported no difference among the study groups in terms of quality of life at 6 months after the surgical operation (data available for 86 patients, EuroQoL 5-level index score 0.90 ± 0.11 vs. 0.89 ± 0.12 for functional testing and angiogram, respectively).

The number of studies was insufficient to perform meaningful secondary analyses or inspection of funnel plots to assess publication bias.

**Figure 4:**
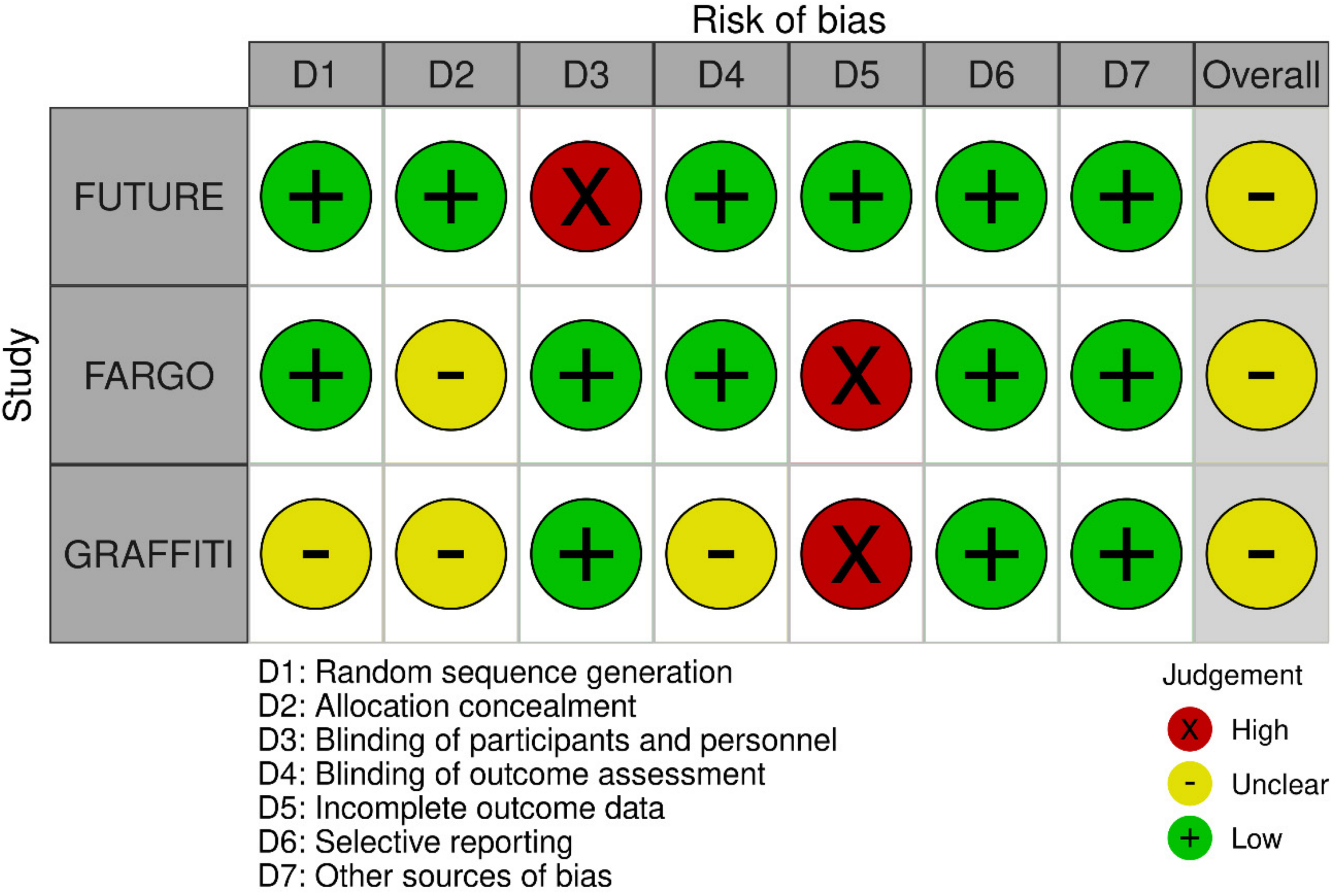
Individual randomised controlled studies risk of bias plot.

**Figure 5:**
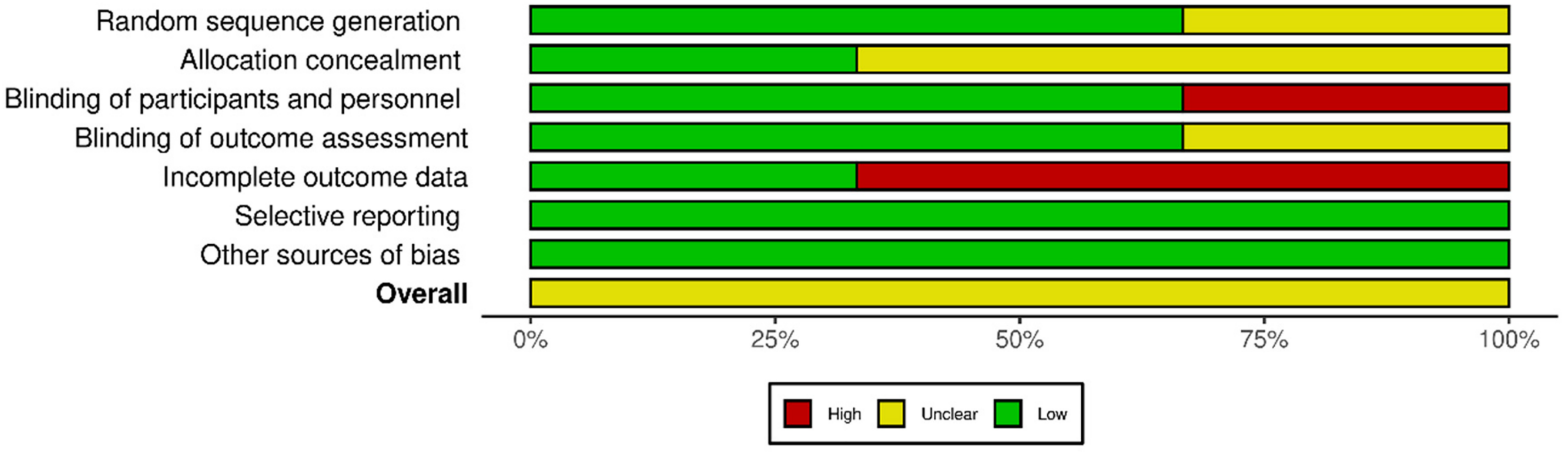
Summary for the randomised controlled studies risk of bias plot.

The quality of the retrieved evidence was poor (Figures 4 and 5). The most critical domain for the RCTs was the blinding of participants and personnel (FUTURE), and the incomplete outcome reporting due to loss of patients at follow-up (FARGO, GRAFFITI). Notwithstanding their intrinsic limitation due to lack of randomisation, observational studies were rated as of adequate quality according to the Newcastle–Ottawa scale (Supplementary Appendix S2).

### 3.2 Practice survey

**Figure 6:**
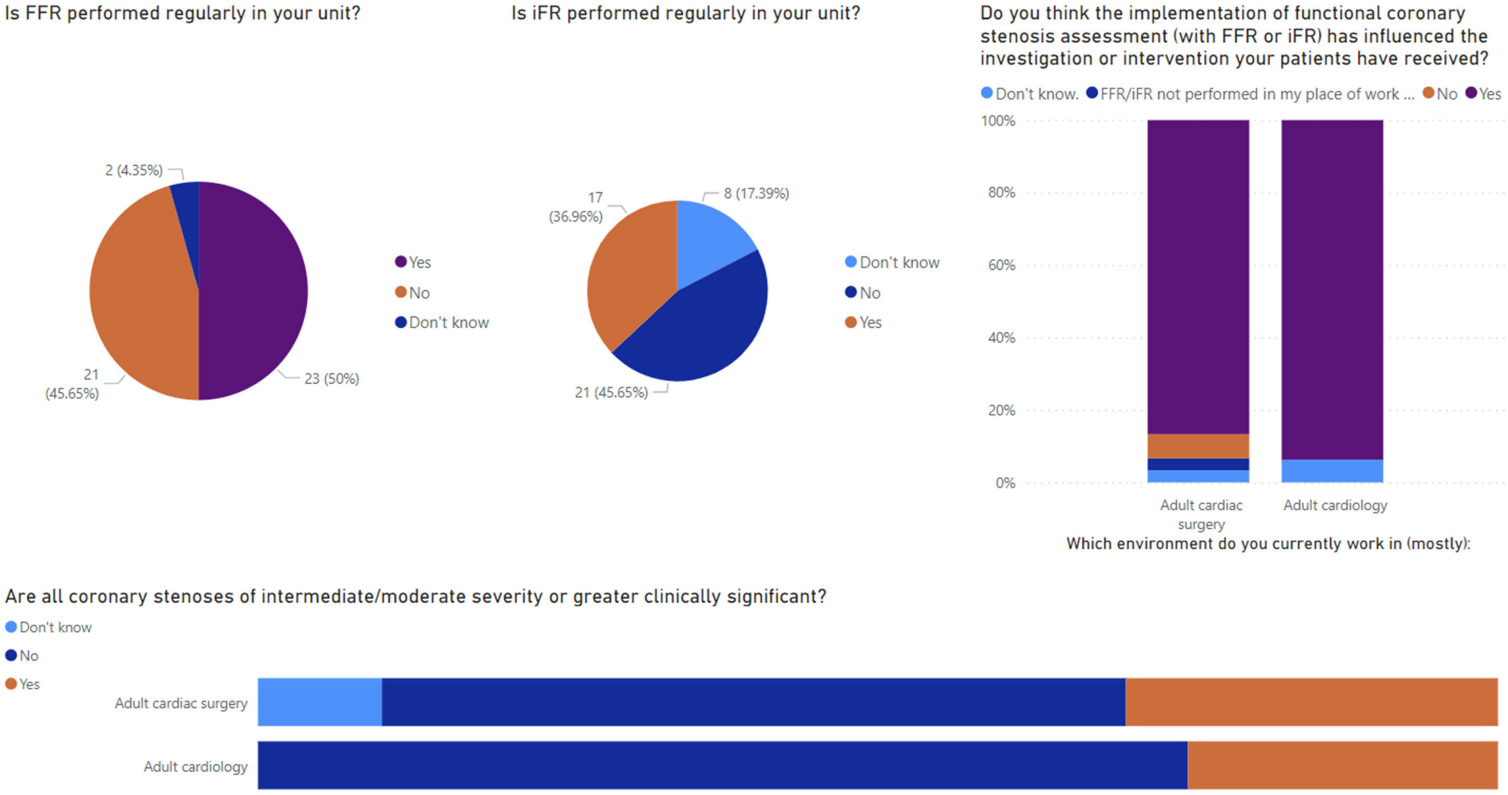
Main results from the clinicians’ survey.

Altogether, 46 responses were received: 30 (65.2%) from those working within adult cardiac surgery and the remaining working within adult cardiology. Most responses were received from consultant (*n* = 16, 34.8%) or registrar level (*n* = 15, 32.6%) surgeons and physicians with the remaining defining themselves as associate specialists, SAS doctors, advanced care practitioners, consultant non-physicians, or “other.” Selected results from the survey are presented in Figure 6.

Within surgical units, 48.4% (*n* = 15) said that FFR was not performed regularly within their unit, and 37.5% (*n* = 6) of cardiologists said the same. The perceived reasons for not offering this service locally included high costs, time constraints, and a lack of adequate training or locally skilled physicians. A small number of responders also noted that there was a perceived paucity of clinical indications resulting in infrequent use and so it is not offered in their unit. Reported rates of alternative functional imaging, primarily non-invasive physiological testing such as CT-FFR, were low; only 14 responders (30.4%) had CT-FFR available to them. In addition, 86.7% (*n* = 26) of surgeons and 75% (*n* = 12) of cardiologists regularly attend an interventional cardiology multidisciplinary team (MDT), with reasons for non-attendance reported as personal scheduling conflicts or lack of local MDT and only regional MDT in large tertiary centres being available.

There were high reported rates of collaboration reported between surgeons and physicians. About 80.6% of surgeons (*n* = 25) discussed coronary angiograms with the reporting physician at least several times per year within their practice. Furthermore, more than half (*n* = 18, 58.1%) have sought a second opinion from a different physician other than the one who conducted the angiogram prior to offering CABG. It was frequently reported by surgeons that this need for rediscussion would likely be reduced if physiological assessments were performed during index coronary angiograms (67.7%, *n* = 21). When asked a similar question, most responding cardiologists agreed that physiological assessment would reduce the need for discussion with a surgeon (83.3%, *n* = 10). Less than half of the cardiologists responding to the survey (41.7%, *n* = 5) seek regular opinions (several times per year or more) from a cardiac surgeon regarding angiogram appearances. Most responders (51.6% *n* = 16 surgeons, 50.0% *n* = 6 cardiologists) believe anatomical and physiological considerations are equally important when considering the outcome of angiography, although similar proportions of surgeons (38.7%, *n* = 12) and cardiologists (41.7%, *n* = 5) believe anatomical considerations were more important than physiological considerations. Further to this, when asked whether all coronary stenoses of intermediate or moderate severity or greater are clinically significant, 58.1% (*n* = 18) of surgeons and 75% (*n* = 12) of cardiologists said no. When asking about the impact of physiological assessments on patient care, 83.4% of surgeons (*n* = 26) and 93.8% of cardiologists (*n* = 15) felt that implementation of functional coronary assessment had influenced the overall investigation or intervention their patients had received since implementation in their unit. We then asked all responders in what way they believe FFR or iFR measurement has influenced their patient's treatment, compared with traditional angiography in isolation. There was heterogeneity of responses and no consensus agreement. Responders noted contrasting experiences, stating their beliefs that physiological assessments resulted in both greater and fewer overall investigations, a greater chance of treatment deferral as well as expediting of the intervention, and both more and less frequent referral for PCI and for CABG. Among surgeons, there was no consensus on how this impacted the intraoperative course with a perceived reduction and increase of number of bypass grafts performed following physiological assessments preoperatively. Finally, we noted from the responses that there was recognition that in the right context, functional coronary testing was beneficial to patients. The limitations of its use are due to time constraints and availability of the testing. More so than invasive testing, non-invasive functional imaging, i.e. CT-FFR, was described as still in its burgeoning stages and not widely available to many heart team members at present. Moreover, there were high reported rates of MDT attendance and cross-specialty communication suggesting that heart team working is generally wide-spread, which is expected given its recommendation by all major guidelines on the management of coronary heart disease (33, 34).

## 4 Discussion

The role of FFR or iFR in guiding surgical strategy remains unclear with conflicting evidence existing. Although we attempted to study the role of both, the evidence for IFR-based strategies in surgical revascularisation is insufficient and thus its role was not assessed.

In this meta-analysis, we focused on RCTs and observational studies only with complete data on patients undergoing CABG both prior to surgery and at follow-up, to obtain measurable results, thus significantly restricting the number of studies. This led to only three trials and four observational studies being included.

Pooled data from RCTs and registries demonstrated no difference in mortality rates (*p* = 0.273) and in myocardial injury (*p* = 0.111) between patients who underwent CABG guided by traditional coronary angiography and those guided by invasive functional assessment (FFR/iFR), number of grafts (*p* = 0.328), and need for repeat unplanned revascularisation (*p* = 0.874).

In our analysis, there was no increase in neurological events (*p* = 0.287) in the physiology-guided CABG group.

In FUTURE (28), the authors tested with a randomised controlled trial whether an FFR-guided treatment strategy was superior to a traditional strategy without FFR (performed for stenoses above 50%) for patients with multivessel coronary disease. The trial was stopped early due to safety concerns, but the results showed no significant difference in major adverse cardiac or cerebrovascular events (MACEs) between the two groups (hazard ratio: 0.97; 95% CI: 0.69–1.36; *p* = 0.85). The FFR group did have a lower rate of revascularisation (*p* = 0.02).

In FARGO (29), the authors assessed in a randomised controlled trial the value of FFR evaluation of coronary artery stenosis (with a visual narrowing of more than 50%) in CABG. The authors found that FFR-guided CABG had similar graft failure rates (16% in the FFR group vs. 12% in the conventional angiography group; *p* = 0.97) and clinical outcomes as angiography-guided CABG. However, FFR was reduced significantly after 6 months in deferred lesions.

Similarly in GRAFFITI (30), angiography-guided and fractional flow reserve (performed for intermediate lesions)-guided CABG were compared. The study found that FFR-guided CABG does not improve graft patency after 1 year (80% in the angiography-guided group vs. 81% in the FFR-guided group, respectively; *p* = 0.885), but it does result in a simplified surgical procedure.

Interestingly, observational studies seemed to produce results that favoured functional tests. In their study, Botman et al. (24) found that bypass grafts on functionally significant lesions were more likely to be patent than bypass grafts on functionally non-significant lesions (8.9% occluded on functionally significant lesions vs. 21.4% on functionally non-significant lesions, *p* < 0.001). Fournier et al. (25) presented a 6-year follow-up study of FFR-guided vs. angiography-guided CABG surgery. The authors found that FFR-guided CABG surgery was associated with a lower rate of death or myocardial infarction at 6 years (hazard ratio 0.59, 95% CI 0.38–0.93; *p* = 0.020). The article also noted that FFR-guided CABG surgery was associated with a lower number of graft anastomoses, a lower rate of on-pump surgery, and a higher graft patency rate. Outcomes were reported at a median duration of 85 months and from angiographic evaluation, the longest of all included studies. Summarising their outcome data, the authors report significantly reduced occlusion rates with FFR-guided compared with angiography-guided grafts (log rank, 0.027, *p* = 0.022). However, only 27.3% (171 of 627) of the evaluated population underwent repeat angiographic assessment.

Glineur et al. (26) studied the impact of preoperative fractional flow reserve on arterial bypass graft anastomotic function to evaluate the use of FFR to guide revascularisation decisions. The authors investigated whether preoperative FFR measurement of coronary lesions is associated with improved patency 6 months after surgical revascularisation using a multiarterial grafting strategy. They found a significant association between the preoperative FFR measurement of the target vessel and the anastomotic functionality at 6 months (FFR AUC 0.92; 95% CI 0.87–0.96 vs. degree of stenosis AUC 0.57, 95% CI 0.48–0.66; *p* < 0.001). The authors concluded that integration of FFR measurement into the preoperative diagnostic workup before multiarterial coronary surgical revascularisation leads to improved anastomotic graft function. In their work from 2018, Moscona et al. (27) compared an anatomical with a physiological assessment of moderate (40%–70%) coronary lesions. They found that the physiological assessment group had a higher rate of complete revascularisation (three vessel anastomoses in 85.7% of the FFR/iFR-guided group vs. 74.7% in the angiography-guided group, *P* < 0.05). The authors concluded that physiological assessment can effectively guide CABG surgery.

Despite the surgeons being blinded to FFR in FARGO, GRAFFITI, and Botman and Glineur, the number of grafts performed was similar in functional-guided CABG compared with the angiography-guided group, with no need for further unplanned revascularisation. This is in contrast to what would be expected from physiology-guided revascularisation trials, as seen in FFR-guided PCI (8). However, patients enrolled in these studies also presented with chronic total occlusion, accounting for 10% in the FUTURE trial, with a mean SYNTAX score of 19 (28), and patients not suitable for PCI (24). It is likely that the complexity of coronary artery disease, with fewer moderate ambiguous lesions and their anatomical locations might have driven revascularisation on an angiography-based strategy mitigating the benefits of functional assessment.

Most trials of functional assessment on coronary lesions are performed with FFR. However, in clinical practice iFR is quicker to perform and has the advantage of not needing hyperaemia, thus avoiding adenosine-related contraindications and unpleasant effects for patients, and providing a faster assessment in the setting of multivessel disease. As proved in the DEFINE—FLAIR and in the SWEDEHEART trials, iFR revascularisation strategy is not inferior to FFR-guided treatment in patients with stable and unstable coronary disease (5) and it has been recently proposed as the index of choice for pressure-based assessment of intermediate lesions (35).

The main findings from our survey of experts in the specific context of patients being considered for CABG surgery are as follows: (a) a perceived low use of physiological assessment both by cardiac surgeons and cardiologists, (b) in a cohort of specialists with high rate of multidisciplinary meeting attendance and discussion; (c) importance of functional and anatomical considerations taken together, with a great proportion of participants considering anatomical features more relevant over functional assessment; (d) with an overall perceived benefit from implementing FFR/iFR in patient care.

Multiple survey responders supported the need for thorough clinical assessment of patients with an emphasis on elucidating symptoms in maximal detail to best stratify patient risk and to identify patients with the greatest perceived benefit from physiological coronary testing. This finding is aligned with the importance of symptoms assessment in patients with stable coronary disease as highlighted in the ORBITA trial (36).

RCTs focusing on the effect of functional assessment of intermediate coronary lesions in patients with stable coronary disease undergoing CABG are needed to clarify the role of these tools to improve outcomes including MACEs, choice of conduit, revascularisation strategy, and long-term graft patency. In addition, the role of functional assessment in the context of guiding revascularisation in patients needing concomitant valve surgery, although not assessed within this study, should be explored in future given the frequency of this presentation in clinical practice.

### 4.1 Limitations

We acknowledge several limitations in this work, which are due to the available evidence and the nature of our investigation. As this work is the result of the analysis of published series, small-sample size, inconsistency in outcome definition, the limited number of randomised studies, and differences in surgical practices and materials adopted during the angiographic assessment may limit the certainty of the evidence. We also acknowledge that while no studies met the predefined threshold of significant heterogeneity, methodological diversity exists between the included studies and that this may contribute low level bias to the findings of our analysis. Patients with concomitant valve lesions, a common population encountered in regular practice, were also excluded in the studies analysed.

Follow-up duration and protocol varied significantly among all included studies that forwent meta-analysis of long-term outcomes. A key limitation in quality of the trials was the incomplete outcomes reporting due to significant loss of patients at follow-up with only 75% and 64% of data available for the primary endpoint of graft patency for the FARGO and GRAFFITI trials, respectively, probably underestimating the effects observed. However, regarding clinical outcomes at follow-up, this was 100% and 98% for both trials, respectively. The FUTURE trial was a non-blinded trial that was stopped prematurely owing to safety concerns regarding increased rate of death in the FFR group, which may have underpowered a difference in a complex population (37). The complexity of the populations included in these studies might have determined increased mortality rates and myocardial ischaemia *per se*.

Another possible confounding factor is that all studies included patients with recent acute coronary syndrome (ACS). Coronary revascularisation guided by physiological principles is endorsed by clinical guidelines but has been confirmed through trials in patients with mostly stable ischaemic heart disease. The role of FFR in ACS for PCI is still controversial as reported in the FLOWER-MI trial and in the FUTURE trial and so the generalisability of these results upon a population of patients with stable angina is unclear (38).

Finally, the retrospective registries included in our study reported a significant higher rate of graft patency in the group receiving physiology-guided revascularisation compared with the angiography only group. However, this was not included as an endpoint of our study, and may be a product of selection bias, hence deriving from the intrinsic limitations of the study design.

## 5 Conclusion

In summary, we believe that physiology assessment should be used as a complementary tool in moderate lesions of uncertain functional significance. In patients undergoing PCI, this would mean defer PCI in case of negative FFR and avoid possible complications related to not necessary PCI. In CABG, data available are still controversial. However, for the anatomical complexity of the setting of microvascular disease (MVD) and in complex patients, probably angiography only still gives the best results. In patients with stable CAD, with low anatomical complexity, physiological tools may guide the revascularisation at multiple stages. Further RCTs with selected populations and anatomical characterisation of the coronary disease are needed to elucidate the role of physiological assessment prior to CABG.

## Data Availability

The raw data supporting the conclusions of this article will be made available by the authors, without undue reservation.
