# Supplementary material for: Fractional flow reserve and instantaneous wave-free ratio in coronary artery bypass grafting: a meta-analysis and practice review
Source: Front Cardiovasc Med. 2024 Mar 7;11:1348341. doi: 10.3389/fcvm.2024.1348341 (PMC10955066; doi:10.3389/fcvm.2024.1348341)
Supplement: Supplementary file 1 [file Datasheet1.docx]

**Supplementary Material**

Appendix 1. Search Strategy for Ovid MEDLINE, adapted for other databases

Ovid MEDLINE(R) ALL <1946 to June 02, 2023>

1 exp Coronary Artery Bypass/

2 (Coronary Artery Bypass or CABG).mp.

3 Fractional Flow Reserve.mp.

4 exp Fractional Flow Reserve, Myocardial/

5 FFR.mp.

6 iFR.mp.

7 instantaneous wave-free ratio.mp.

8 'controlled study'/exp OR 'prospective study'/exp OR cohort analysis/exp OR cohort.ti,ab OR 'compared'.ti,ab OR 'groups'.ti,ab OR 'case control'.ti,ab OR 'multivariate'.ti,ab

9 1 or 2

10 3 or 4 or 5 or 6 or 7

11 8 and 9 and 10

Appendix 2 – Risk of bias assessment for the observational studies included according to the Newcastle-Ottawa scale

|  | **Representativeness of the exposed cohort** | | **Selection of the non-exposed cohort** | | **Ascertainment of exposure** | | **Demonstration that outcome of interest was not present at start of study** | | **Comparability of cohorts on the basis of the design or analysis controlled for confounders** | | **Assessment of outcome** | | **Was follow-up long enough for outcomes to occur?** | | **Adequacy of follow-up of cohorts** | |
| --- | --- | --- | --- | --- | --- | --- | --- | --- | --- | --- | --- | --- | --- | --- | --- | --- |
| **Botman 2007** | **1** | **A** | **1** | **A** | **1** | **A** | **1** | **A** | **2** |  | **1** | **B** | **1** | **A** | **1** | **B** |
| **Fournier 2018 (A)** | **1** | **A** | **1** | **A** | **1** | **A** | **1** | **A** | **2** |  | **1** | **B** | **1** | **A** | **1** | **A** |
| **Glineur 2019** | **1** | **A** | **1** | **A** | **1** | **A** | **1** | **A** | **2** |  | **1** | **A** | **1** | **A** | **1** | **B** |
| **Moscona 2018** | **0** | **C** | **1** | **A** | **1** | **A** | **1** | **A** | **1** | **A** | **1** | **B** | **1** | **A** | **1** | **A** |

Appendix 3 – Outline of the online survey

1. Which environment do you currently work in:
   1. Cardiac surgery
   2. Cardiology
2. Current role (select most appropriate)
   1. Consultant physician or surgeon
   2. Associate Specialist
   3. Registrar (training or non-training grade)
   4. SAS doctor
   5. Consultant non-physician, non-surgeon ie. Consultant nurse
   6. Non-consultant nurse specialist
   7. Advanced care practitioner, Advanced nurse practitioner
   8. Other (free text)
3. Do you regularly attend an interventional cardiology MDT?
   1. Yes
   2. No
      1. If no, why not? (free text)
4. Do you perform, or have an active role in supporting the performance of, coronary angiography?
   1. Yes
   2. No
5. Do you perform, or have an active role supporting the performance of, FFR?
   1. Yes
   2. No
6. Do you perform, or have an active role supporting the performance of, iFR?
   1. Yes
   2. No
7. Which of these, if any, are performed in your unit? Select all which apply.
   1. FFR
   2. iFR
   3. CT Cardiac calcium scoring
   4. CT-Coronary Angiography
   5. CT-FFR
8. Is FFR performed regularly (‘regularly’ defined as at least for the majority of indeterminate lesions identified during angiography) in your unit?
   1. Yes
   2. No
   3. Don’t know
9. If no, what are the biggest limitations to the use of FFR in your department? Select all which apply
   1. Cost.
   2. Time constraints.
   3. Lack of adequate training/skills within local team.
   4. Inadequate frequency for its use/too infrequent clinical indication
   5. Lack of departmental support for its clinical accuracy/utility.
   6. Determined to post an unacceptable clinical risk to patients.
   7. Other (free text)
   8. Not applicable, I am a member of cardiac surgery team.
10. Is iFR performed regularly (‘regularly’ defined as at least for the majority of indeterminate lesions identified during traditional digital subtraction angiography) in your unit?
    1. Yes
    2. No
    3. Don’t know
11. If no, what are the biggest limitations to the use of iFR in your department? Select all which apply
    1. Cost.
    2. Time constraints.
    3. Lack of adequate training/skills within local team.
    4. Inadequate frequency for its use/too infrequent clinical indication
    5. Lack of departmental support for its clinical accuracy/utility.
    6. Determined to post an unacceptable clinical risk to patients.
    7. Other (please specify in free text at end of survey)
    8. Not applicable, I am a member of cardiac surgery team.
12. Do you think the functional significance of coronary stenosis is important to establish?
    1. Yes
    2. No
13. Do you think functional coronary stenosis assessment (with FFR or iFR) has influenced the intervention your patients have received in the past?
    1. Yes
    2. No
    3. Unsure
14. If yes, compared to traditional angiography alone, how do you think FFR/iFR measurement has influenced your patients’ treatments? Select all which apply.
    1. More likely to receive further investigations.
    2. Less likely to receive further investigations.
    3. More likely to receive PCI interventions.
    4. Less likely to receive PCI interventions.
    5. More likely to be referred for CABG.
    6. Less likely to be referred for CABG.
    7. More likely to have treatment (of any kind) deferred (performed at a later date/urgency of intervention downgraded)
    8. More likely to have treatment (of any kind) expedited (performed more quickly).
    9. I do not think functional tests influence patient care.
15. Compared to having “traditional angiography” alone, how do you think FFR calculations have impacted patients undergoing CABG (if at all)? Select all which apply.
    1. More likely to be referred for CABG following FFR measurement.
    2. Less likely to be referred for CABG following FFR measurement.
    3. No impact on rate of referral for CABG following FFR measurement.
    4. Receive more bypass grafts during CABG following FFR measurement.
    5. Receive fewer bypass grafts during CABG following FFR measurement.
    6. More likely to have treatment (of any kind) deferred (performed at a later date/urgency of intervention downgraded)
    7. More likely to have treatment (of any kind) expedited (performed more quickly).
    8. No impact on number of bypass grafts during CABG following FFR measurement.
    9. I do not think FFR measurement has any significant impact on intervention referred or received.
16. Compared to having “traditional angiography” alone, how do you think iFR calculations have impacted patients undergoing CABG (if at all)? Select all which apply.
    1. More likely to be referred for CABG following iFR measurement.
    2. Less likely to be referred for CABG following iFR measurement.
    3. No impact on rate of referral for CABG following iFR measurement.
    4. Receive more bypass grafts during CABG following iFR measurement.
    5. Receive fewer bypass grafts during CABG following iFR measurement.
    6. No impact on number of bypass grafts during CABG following iFR measurement.
    7. I do not think iFR measurement has any significant impact on intervention referred or received.
17. What steps do you think need to be taken within your specialty to improve DIAGNOSIS of significant coronary stenosis?

[free text]

1. What steps do you think need to be taken within your specialty to improve TREATMENT of significant coronary stenosis?

[free text]

1. What are the areas of uncertainty or controversy regarding FFR or iFR that you would like to see addressed in future research?

[free text]

1. [surgeons] How often do you have to seek a second opinion from a cardiologist in regard to the report of a coronary angiogram?
   1. Never
   2. Rarely
   3. Sometimes
   4. Often
   5. Very often
2. [cardiologist] How often are you asked for a second opinion from a cardiothoracic surgeon in regard to the report of a coronary angiogram?
   1. Never
   2. Rarely
   3. Sometimes
   4. Often
   5. Very often
3. Do you think anatomical considerations take priority over physiological assessment in your decision-making?
   1. Yes
   2. No
   3. Unsure
4. Any other comments about FFR or iFR? (free text)

[free text]
